# Year-round West Nile Virus Activity, Gulf Coast Region, Texas and Louisiana

**DOI:** 10.3201/eid1009.040203

**Published:** 2004-09

**Authors:** Robert B. Tesh, Ray Parsons, Marina Siirin, Yvonne Randle, Chris Sargent, Hilda Guzman, Taweesak Wuithiranyagool, Stephen Higgs, Dana L. Vanlandingham, Adil A. Bala, Keith Haas, Brian Zerinque

**Affiliations:** * University of Texas Medical Branch, Galveston, Texas, USA;; † Harris County Public Health and Environmental Services, Houston, Texas, USA;; ‡ Galveston County Mosquito Control District, Dickinson, Texas, USA;; § Mosquito Control Contractors, Inc., New Iberia, Louisiana, USA

**Keywords:** West Nile virus, arbovirus, viral diseases, vector-borne disease, virus ecology, infectious diseases, virus persistence, Culex mosquitoes, dispatch

## Abstract

West Nile virus (WNV) was detected in 11 dead birds and two mosquito pools collected in east Texas and southern Louisiana during surveillance studies in the winter of 2003 to 2004. These findings suggest that WNV is active throughout the year in this region of the United States.

Since the initial recognition of West Nile virus (WNV) in North America in 1999 (*1*), one question that has perplexed epidemiologists and public health officials is how the virus persists during the winter in temperate regions. Arbovirologists and vector biologists have long pondered how arboviruses are maintained during periods when their vectors are absent or inactive (*2**–**5*).

For WNV, little information is available on how the virus is maintained in North America during cold periods, when little or no adult mosquito activity occurs. In the winter after the initial 1999 West Nile outbreak in the northeastern United States, Nasci et al. (*6*) reported detecting West Nile viral RNA and infectious virus in hibernating adult *Culex* mosquitoes collected from underground sanitation tunnels, vacant buildings, and other protective structures in New York City. During the same month (February 2000), another group (*7*) reported isolating WNV from tissues of a freshly dead hawk found in Westchester County just north of the city. These two observations suggested that, in northern latitudes, WNV may be maintained locally in hibernating *Culex* mosquitoes, as demonstrated earlier for St. Louis encephalitis virus (*8*), but that low-level virus transmission may also occur during winter.

## The Study

WNV was initially detected in the Houston metropolitan area (Harris County) in the summer of 2002; 105 confirmed human infections with WNV were reported in Houston during the first year (*9*). In addition, 307 WNV isolates were obtained from dead birds, and 851 WNV-positive pools of *Cx. pipiens quinquefasciatus* were collected during our surveillance studies in the summer and fall of 2002 (*9*). In 2003, we began a long-term study on the ecology of WNV in Harris County. As part of this program, mosquito and dead bird collections were made by Harris County mosquito control personnel throughout the year. Mosquito collections were made at selected sites throughout the county, with CDC-type light traps and gravid traps (*9*). Our trapping methods were designed to sample mainly *Cx. p. quinquefasciatus*; this species represented >95% of the mosquitoes collected. After collection, mosquitoes were sorted into pools of <50 females (mean pool size 28.8) and assayed for WNV by an antigen-capture enzyme immunoassay (EIA) (*9*). Selected EIA-positive mosquito pools (including all winter positives) were confirmed by reverse transcription–polymerase chain reaction (RT-PCR), as described previously (*10*).

After the initial detection of WNV in Houston in 2002, the Harris County mosquito control personnel established a dead bird surveillance system (*9*); media reports and public messages instructed county residents to report dead birds, most of which were picked up by the county mosquito control personnel. After collection and species identification, the bird carcasses were frozen at –75°C for subsequent transport to the University of Texas Medical Branch in Galveston, where a sample of brain from each dead bird was cultured for WNV. Culture methods and tests used for virus confirmation were described earlier (*9**,**10*). During 2003 and January 2004, the University of Texas Medical Branch group also received a few dead birds from mosquito control districts in Galveston County, Texas, and Louisiana. These avian samples were processed as described above, but are listed separately from the Harris County collections.

## Conclusions

Table 1 summarizes the monthly WNV surveillance results for dead birds and mosquitoes tested from Harris County from January 2003 to March 2004. During this 15-month period, 1,623 dead birds, representing 83 avian species, were examined. The number and species of dead birds examined each month varied, reflecting seasonal changes in local avian abundance and deaths, interest of the local citizens in reporting dead birds, winter bird migration into the region, and limits in our ability to process samples. For example, in June, July, and August 2003, a total of 3,352 dead birds were reported by local residents to the county mosquito control staff. During this period, we limited the number of birds tested for WNV to approximately 50 per week, and an attempt was made to sample birds from a variety of different sites within the county. Thus in these 3 months, only 588 dead birds (approximately 17% of the total reported for the period) were actually tested. Also during this period, corvids (Blue Jays and crows) were preferentially selected to be tested, since our experience indicated that these species were most likely to yield virus (*9*). In contrast, during the winter months (November, December, January, February, and March) fewer dead birds were reported; most of these birds were tested, regardless of species or collection locality. For this reason, Blue Jays (the most common species sampled) represented 35.9% of the birds tested during June, July, and August 2003 but only 7.7% of the birds tested during the months of November, December, January, February, and March.

**Table 1:** Monthly summary of dead birds and mosquitoes tested for West Nile virus (WNV) in Harris County, Texas

Year/month	Mean temperature (°C)^a^	Total birds tested	No. WNV-positive (% positive)	Total mosquitoes tested	No. WNV-positive mosquito pools^b^	WNV minimum infection rate
2003						
January	10.6	46	0	2,164	0	0.00
February	12.5	62	0	1,146	0	0.00
March	16.7	80	0	5,304	0	0.00
April	21.4	98	1 (1.0)	39,000	0	0.00
May	27.1	213	4 (1.9)	58,698	2	0.03
June	27.9	219	33 (15.1)	42,041	40	0.95
July	28.2	205	72 (35.1)	54,582	203	3.72
August	28.9	164	83 (50.6)	40,184	128	3.19
September	25.5	127	38 (29.9)	34,691	21	0.61
October	22.2	73	11 (15.1)	41,465	4	0.10
November	18.7	20	2 (10)	7,562	2	0.13
December	12.6	7	1 (14.3)	8,411	0	0.00
2004						
January	13.2	26	1 (3.8)	12,816	0	0.00
February	12.3	146	3 (2.1)	9,790	0	0.00
March	19.5	137	1 (0.7)	14,714	0	0.00
Total	–	1,623	250 (15.4)	372,568	400	1.07

^a^Mean monthly temperature in Houston (Harris County) (*11*). ^b^Mean pool size was 28.2 mosquitoes/pool.

A similar seasonal bias occurred in our mosquito sampling. During the hottest months of the year (June, July, and August) in Harris County, large numbers of mosquitoes were collected, exceeding our capacity to test them. Thus only a subsample of the mosquitoes collected during this period were tested for WNV. In contrast, during the winter months, adult *Cx. p. quinquesfasciatus* abundance and activity were markedly reduced. During this period, most of the mosquitoes that were collected in traps were assayed for WNV. Our results provide information about the seasonal pattern of WNV activity in the western Gulf region, despite the sampling bias.

Table 2 shows the species composition and WNV infection rates of dead birds collected in Harris County from January 2003 to March 2004. Overall, Blue Jays were the most common dead birds submitted for testing and represented 23.2% of the total; 48.9% of the dead Blue Jays yielded virus. Only 23 American Crows were submitted for WNV testing, but 16 (69.6%) of them were virus-positive upon culture. Crows are much less abundant than Blue Jays in urban areas of the county. Mourning Doves were another commonly submitted dead bird (17.1% of total), but only 2.2% of this species yielded WNV after culture.

**Table 2:** West Nile virus–species infection rates among 248 culture-positive dead birds collected in Harris County, Texas, January 2003–March 2004

Common name	Scientific name	Total tested	No. infected (%)
Blue Jay	*Cyanocitta cristata*	376	184 (48.9)
American Crow	*Corvus brachyrhynchos*	23	16 (69.6)
Loggerhead Shrike	*Lanius ludovicianus*	14	7 (50)
House Sparrow	*Passer domesticus*	119	19 (16.0)
Northern Mockingbird	*Mimus polyglottus*	99	8 (8.1)
Mourning Dove	*Zenaida macroura*	278	6 (2.2)
Rock Dove	*Columba livia*	48	1 (2.1)
Inca Dove	*Columbina inca*	38	1 (2.6)
Great-crested Flycatcher	*Myiarchus crinitus*	1	1 (100)
Carolina Chickadee	*Parus carolinensis*	3	1 (33.3)
Tufted Titmouse	*Baeolophus bicolor*	2	1 (50)
Common Grackle	*Quiscalus quiscula*	84	3 (3.6)
Orchard Oriole	*Icterus spurius*	3	1 (33.3)
American Goldfinch	*Carduelis tristis*	1	1 (100)

As indicated in Table 1, most of the WNV-positive dead birds and mosquitoes from the county were collected during the summer months of June, July, and August. These months are also the three warmest in Harris County (*11*). However, WNV was also detected in birds and mosquitoes during most other months. Table 3 summarizes the WNV-positive samples identified in our laboratories from November 2003 to March 2004. Ten WNV-positive bird or mosquito samples were from Harris County; the other three positive dead bird samples were submitted from Galveston County, Texas, and Iberia Parish, Louisiana. These isolations of WNV from dead birds and the identification of viral RNA in physiologically active adult mosquitoes collected during the winter season (November–March) imply that the virus is active year-round in Harris County (Table 1) and the western Gulf region. The wide geographic distribution of localities yielding infected birds and mosquitoes also suggests that virus was not restricted to a single community or site but was widespread. Our data from Harris County indicate that peak virus activity occurred mainly during the warm months of the year (June–September), as observed elsewhere in North America (*12*), but that low-level virus activity continued during the rest of the year in this region. Prolonged periods with temperatures <0°C are uncommon in Harris County and the western Gulf Coast, so *Cx. p. quinquefasciatus* (the presumed vector of WNV in the region) does not enter a true diapause, as does its northern counterpart, *Cx. pipiens* (*13*). Our field observations in Harris County indicate that *Cx. p. quinquefaciatus* adults become relatively inactive during cold periods, resting under buildings and in storm drains and sewers; however, these mosquitoes become active again during warm periods in the winter months. The fact that adults can be captured in light traps and eggs laid in gravid traps throughout the year in the county (Table 1) is evidence of their continual activity. This intermittent host-seeking activity throughout the winter probably accounts for continued low-level WNV transmission and infection in the resident avian population. On the basis of these observations, we believe that this mechanism is probably the principal one by which WNV overwinters and persists in the western Gulf region of the United States.

**Table 3:** Confirmed West Nile virus (WNV) activity in birds and mosquitoes collected in east Texas and southern Louisiana

Date collected	WNV-positive sample	Locality
Nov. 3, 2003	Blue Jay	Santa Fe, Galveston Co., TX
Nov. 7, 2003	Blue Jay	Dickinson, Galveston Co., TX
Nov. 14, 2003	Blue Jay	Houston, Harris Co., TX
Nov. 18, 2003	Blue Jay	Pasadena, Harris Co., TX
Nov. 20, 2003	*Culex p. quinquefasciatus* pool	Houston, Harris Co., TX
Nov. 20, 2003	*Cx. p. quinquefasciatus* pool	Houston, Harris Co., TX
Dec. 16, 2003	American Crow	Spring, Harris Co., TX
Jan. 2, 2004	Northern Cardinal	New Iberia, Iberia Parish, LA
Jan. 22, 2004	Blue Jay	Tomball, Harris Co., TX
Feb. 12, 2004	American Goldfinch	Houston, Harris Co., TX
Feb. 12, 2004	American Crow	Kingwood, Harris Co., TX
Feb. 19, 2004	Loggerhead Shrike	Houston, Harris Co., TX
Mar. 4, 2004	American Crow	Kingwood, Harris Co., TX

The results of our study also confirm observations by others (*14*) that surveillance of dead birds is a sensitive method for detecting early WNV activity. The use of sentinel animals (in this case dead birds) is a well-established method of arbovirus surveillance and sometimes detects virus activity during periods when none can be detected in mosquitoes (*15*). The presumed increased sensitivity of dead bird surveillance may explain why a few bird isolates of WNV were obtained each month during winter, but no virus activity was detected in *Cx. p. quinquefaciatus* during the same period.
